# Multicenter Evaluation of Diagnostic Circulating Biomarkers to Detect Sight-Threatening Diabetic Retinopathy

**DOI:** 10.1001/jamaophthalmol.2022.1175

**Published:** 2022-05-05

**Authors:** Sarega Gurudas, Karen Frudd, Jayapal Jeya Maheshwari, Yeddula Rebecca Revathy, Sobha Sivaprasad, Shruthi Mahalakshmi Ramanathan, Vignesh Pooleeswaran, A. Toby Prevost, Eleni Karatsai, Sandra Halim, Shruti Chandra, Paul Nderitu, Dolores Conroy, Subramanian Krishnakumar, Sowmya Parameswaran, Kuppamuthu Dharmalingam, Kim Ramasamy, Rajiv Raman, Colin Jones, Haralabos Eleftheriadis, John Greenwood, Patric Turowski

**Affiliations:** 1 Institute of Ophthalmology, University College London, London, United Kingdom; 2 Aravind Medical Research Foundation, Proteomics Department, No.1 Anna Nagar, Madurai, India; 3 Vision Research Foundation, Chennai, India; 4 National Institute of Health Research Moorfields Biomedical Research Centre, Moorfields Eye Hospital London NHS Foundation Trust, London, United Kingdom; 5 Nightingale-Saunders Clinical Trials and Epidemiology Unit, King’s College London, London, United Kingdom; 6 Ophthalmology Department, King’s College University Hospital Trust, London, United Kingdom; 7 Aravind Eye Hospital, Madurai, India; 8 Norfolk and Norwich University Hospitals NHS Trust, Norwich, United Kingdom

## Abstract

**Importance:**

It is a global challenge to provide regular retinal screening for all people with diabetes to detect sight-threatening diabetic retinopathy (STDR).

**Objective:**

To determine if circulating biomarkers could be used to prioritize people with type 2 diabetes for retinal screening to detect STDR.

**Design, Setting, and Participants:**

This cross-sectional study collected data from October 22, 2018, to December 31, 2021. All laboratory staff were masked to the clinical diagnosis, assigned a study cohort, and provided with the database containing the clinical data. This was a multicenter study conducted in parallel in 3 outpatient ophthalmology clinics in the UK and 2 centers in India. Adults 40 years and older were categorized into 4 groups: (1) no history of diabetes, (2) type 2 diabetes of at least 5 years’ duration with no evidence of DR, (3) nonproliferative DR with diabetic macular edema (DME), or (4) proliferative DR. STDR comprised groups 3 and 4.

**Exposures:**

Thirteen previously verified biomarkers were measured using enzyme-linked immunosorbent assay.

**Main Outcomes and Measures:**

Severity of DR and presence of DME were diagnosed using fundus photographs and optical coherence tomography. Weighted logistic regression and receiver operating characteristic curve analysis (ROC) were performed to identify biomarkers that discriminate STDR from no DR beyond the standard clinical parameters of age, disease duration, ethnicity (in the UK) and hemoglobin A_1c_.

**Results:**

A total of 538 participants (mean [SD] age, 60.8 [9.8] years; 319 men [59.3%]) were recruited into the study. A total of 264 participants (49.1%) were from India (group 1, 54 [20.5%]; group 2, 53 [20.1%]; group 3, 52 [19.7%]; group 4, 105 [39.8%]), and 274 participants (50.9%) were from the UK (group 1, 50 [18.2%]; group 2, 70 [25.5%]; group 3, 55 [20.1%]; group 4, 99 [36.1%]). ROC analysis (no DR vs STDR) showed that in addition to age, disease duration, ethnicity (in the UK) and hemoglobin A_1c_, inclusion of cystatin C had near-acceptable discrimination power in both countries (area under the receiver operating characteristic curve [AUC], 0.779; 95% CI, 0.700-0.857 in 215 patients in the UK with complete data; AUC, 0.696; 95% CI, 0.602-0.791 in 208 patients in India with complete data).

**Conclusions and Relevance:**

Results of this cross-sectional study suggest that serum cystatin C had good discrimination power in the UK and India. Circulating cystatin-C levels may be considered as a test to identify those who require prioritization for retinal screening for STDR.

## Key Points

**Question:**

Can circulating serum biomarkers distinguish people with sight-threatening diabetic retinopathy (STDR) from those with no DR?

**Findings:**

This multicenter cross-sectional study of 538 participants found an incremental benefit of circulating cystatin C beyond the standard clinical variables in discriminating STDR from no DR. Cystatin C outperformed 12 other biomarkers found to be distinguished in STDR in previous research.

**Meaning:**

Results of this study suggest the consideration of circulating cystatin C levels as a triage test in prioritizing people with type 2 diabetes from the community for retinal screening in resource-restricted settings.

## Teaser

This cross-sectional study investigates the use of circulating serum biomarkers to distinguish individuals without diabetic retinopathy from those with sight-threatening diabetic retinopathy.

## Introduction

There are approximately 537 million people living with diabetes globally, the majority of whom have type 2 diabetes.^1^ Timely identification and prompt treatment of sight-threatening diabetic retinopathy (STDR) will reduce the risk of visual impairment in people with diabetes. Therefore, regular retinal screening is recommended, but global coverage of systematic DR screening is a formidable challenge.^2,3^ Approximately 80% of people with diabetes live in low- and middle-income countries where resources are restricted.^1^ Visual impairment owing to STDR is also more common in minoritized ethnic groups.^1^ Therefore, a paradigm shift in DR screening strategy is required.

An alternative and more cost-effective risk-based strategy for early identification of STDR may be possible if circulating biomarkers could be used to triage those at risk of STDR for retinal screening. Previous discovery and verification studies have reported a selection of circulating biomarkers with good diagnostic power to identify those with STDR.^4^ These need to be validated in a large cohort in a clinical setting before being proposed as a viable triage tool for retinal screening.^4^

In this study, we aimed to evaluate previously verified blood biomarkers using enzyme-linked immunosorbent assay (ELISA) for their potential usefulness as indicators of STDR.

## Methods

This study was approved by the National Research Ethics Service in the UK and the institutional review board of Vision Research Foundation and Aravind Medical Research Foundation in India. All patients provided written informed consent, and the study followed the Declaration of Helsinki. Participants did not receive any compensation or incentives to participate. This study followed the Strengthening the Reporting of Observational Studies in Epidemiology (STROBE) reporting guidelines.

### Study Design and Participants

This multicenter cross-sectional study was conducted from October 22, 2018, to December 31, 2021, to evaluate previously verified circulating biomarkers for STDR in adults 40 years and older with type 2 diabetes. The study was conducted in parallel in the UK and India. In the UK, the study population was recruited at 3 outpatient ophthalmology clinics, with 2 in London, UK, and 1 in Norwich, UK. Participant race and ethnicity information was gathered based on self-reported data and classified according to their relevant office for national statistic classification. Race and ethnicity categories included South Asian, other Asian (ie, as categorized by the UK 2011 Census, an individual who is not Bangladeshi, Chinese, Indian, or Pakistani is classified as other Asian), Black, White, and other (ie, any individual ethnicity that is not named in the UK 2011 Census). Prior studies have shown that racial and ethnic minoritized groups are disproportionately affected by STDR.^5^ In India, study participants were recruited at 2 centers and included exclusively people of Indian origin residing in both urban and rural regions in Tamil Nadu, India. Participant sex was determined based on self-reported data.

### Masking

Routinely collected blood parameters such as glycated hemoglobin A_1c_ (HbA_1c_), estimated glomerular filtration rate (eGFR), and lipids were measured in hospital laboratories. The samples were anonymized before transfer to the laboratories. All laboratory staff were masked to the clinical diagnosis and data and were assigned a study cohort.

### Selection of Biomarkers

The selected biomarkers comprised complement factor B (CFB), complement factor H (CFH), serpin A4 (kallistatin), α2 macroglobulin (A2m), cystatin C, thrombin (F2), lipoprotein-associated phospholipase A2 (Lp-PLA2), leucine-rich α2 glycoprotein 1 (LRG-1), 8-hydroxy-2′-deoxyguanosine (8-OHDG), afamin, and apolipoprotein A1 (ApoA1), B (ApoB), and C3 (ApoC3).

### Preparation of Serum Samples

Laboratory researchers in both countries followed the same storage and processing instructions. Blood samples were collected in serum separator tubes (BD vacutainer [BD]), allowed to stand for 60 to 120 minutes, then centrifuged at 1100*g* to 1300*g* for 10 minutes. Serum was then aliquoted, frozen, and stored at −80 °C until sample processing in each laboratory. For the UK cohort, all 13 markers were analyzed in a single University College London laboratory whereas for the Indian cohort, 6 markers were analyzed at the Aravind Eye Hospital, Madurai, India, and 7 at the Vision Research Foundation, Chennai, India.

### Enzyme-Linked Immunosorbent Assay 

Serum samples were thawed on ice and serially diluted to the required, previously identified, concentration. ELISA procedures, including the establishments of standard curves, were carried out in accordance with manufacturer’s instructions (eTable 1 in the Supplement). Final absorbance values were measured on spectrophotometers. Dilutions of known protein standards (as provided by the manufacturers) were included on each plate. From this, standard curves were derived using 4-parameter logistic regression, in GraphPad Prism. Absolute values for each experimental sample were interpolated from these curves.

### Outcome Groups

Group 1 with no diabetes was compared with group 2, which served as the control for this study and consisted of people with type 2 diabetes for at least 5 years and no evidence of any DR. Individuals with STDR consisted of group 3, individuals with type 2 diabetes and nonproliferative DR (NPDR) with diabetic macular edema (DME) or group 4, individuals with type 2 diabetes and PDR with or without DME. DR and DME were diagnosed using fundus photographs and optical coherence tomography, respectively.

### Sample Size

A sample size of 45 participants with diabetes and no DR was sufficient for modeling STDR outcomes as this reliably distinguished between observed specificity estimates of 60%, 75%, and 90%, having respective 95% CI half-widths of 14.3%, 12.7%, and 8.8%. A sample size of 50 participants allowed for an anticipated 10% of missing data. Assuming that 135 of 150 participants (90.0%) recruited with STDR have complete data, observed sensitivity estimates of 60%, 75%, and 90% for detecting STDR would also be well distinguished, having 95% CI half-widths of 5.1%, 7.3%, and 8.3%, respectively. Sample groups with full data of sizes 135 and 45 participants also provide informative 95% CI half-widths of 0.09, 0.07, and 0.04 for observed areas under the receiver operative characteristic (ROC) curve of 0.6, 0.75, and 0.9, respectively.

### Statistical Analyses

Data were summarized by outcome group and by country, using mean (SD) or median (IQR) for continuous variables and number (%) for categorical variables. Statistical differences between no DR (group 2) and no diabetes (group 1) as well as between no DR (group 2) and individual STDR groups 3 and 4 were compared using the *t* test or Mann-Whitney *U* test for continuous variables and χ^2^ test or Fisher exact test for categorical variables. Probability weighted comparisons were made between no DR and combined STDR group. Potential multicollinearity was assessed using the variance inflation factor. Weighted logistic regression was used to identify variables that improve detection of STDR relative to no DR, in addition to a (base) model consisting of known risk factors for STDR: age, disease duration race and ethnicity (in the UK), and HbA_1c_. Owing to the low prevalence of STDR in people with diabetes, recruiting a cohort that is representative of the general population in the proportion with STDR would require a large sample size to ensure there were sufficient events. Therefore, we oversampled STDR groups in our study and used weighted logistic regression to mitigate differences in the sample and population prevalence induced by case-control sampling.^6^ The diabetes population proportions of the groups were derived as follows: group 2 (no DR), 93.96%; group 3 (NPDR with DME), 5.28%; and group 4 (PDR), 0.76% for the UK from the English Diabetes Eye Screening Programme^7^ and group 2 (no DR), 94.80%; group 3 (NPDR with DME), 4.45%; and group 4 (PDR), 0.75% for South India from the South India part of the Statistical and Economic Modeling Study of Risk-Based Stratified and Personalized Screening for Diabetes and its Complications in India (SMART) India study,^8^ after discounting the NPDR without DME group, not recruited in this study. Probability weights w_i_ for each disease group, were calculated as *τ_i_*/*y_i_*, the ratio of the population proportion (*τ_i_*) and the sample proportion (*ȳ_i_*). Variables that reached statistical significance in the adjusted analysis were introduced into a forward stepwise selection routine with an entry criterion of α = 0.1, with age, disease duration, race and ethnicity (in the UK), and HbA_1c_ forced into the models. Log_e_ transformation was used for variables serum creatinine, C-reactive protein, triglycerides, A2m, 8-OHDG, ApoA1, ApoB, ApoC3, ApoB/ApoA1, LRG-1, and cystatin C for logistic regression analysis, to avoid estimating misleading associations from influential outlying observations. Odds ratios (ORs), therefore, are interpreted in relation to relative percentage increase in the biomarker rather than per absolute increase in the measured units for other biomarkers.

The final models were summarized using ORs with 95% CIs and the ROC curve. Sensitivity analysis was carried out to compare the area under the curve (AUC) of closely related biomarkers serum creatinine and cystatin C. In addition, cystatin C levels were compared between STDR (groups 3 and 4) and no DR (group 2) in patients with normal kidney function (eGFR ≥90 mL/min/1.73 m^2^) as elevated cystatin C levels may be attributable to diabetic kidney disease rather than STDR. All 2-tailed *P *values < .10 were considered statistically significant. Statistical analysis was carried out in RStudio, version 3.6.3 (RStudio)^9^ and Stata MP, version 15 (StataCorp).^10^

## Results

### Descriptive Analysis

In total, 629 samples (365 in UK and 264 in India) were collected from October 22, 2018, to December 31, 2021, exceeding the minimum requirement for 500; however, in the UK, 91 biomarker samples were lost owing to freezer failure (eFigure 1 in the Supplement). A total of 538 participants (mean [SD] age, 60.8 [9.8] years; 319 men [59.3%]; 219 women [40.7%]) were recruited into the study. A total of 264 participants (49.1%) were from India (group 1, 54 [20.5%]; group 2, 53 [20.1%]; group 3, 52 [19.7%]; group 4, 105 [39.8%]) all of whom were South Indian, and 274 participants (50.9%) were from the UK (group 1, 50 [18.2%]; group 2, 70 [25.5%]; group 3, 55 [20.1%]; group 4, 99 [36.1%]) with 73 South Asian (26.6%), 20 other Asian (7.3%), 60 Black (21.9%), 112 White (40.9%), and 9 other (3.3%) race and ethnicity (Table 1). In the UK, mean (SD) age of participants was 63.0 (10.5) years and 148 (54.0%) were men, whereas in India, mean (SD) age was 58.4 (8.5) years and 171 (64.8%) were men.

**Table 1.** Summary Demographic, Clinical, and Biomarker Data of the Study Sample by Country and Outcome Group

Variable	United Kingdom							India						
	PDR ± DME (n = 99) [total No.]	NPDR + DME (n = 55) [total No.]	No DR (n = 70) [total No.]	No diabetes (n = 50) [total No.]	*P* value ^a^	*P* value ^b^	*P* value ^c^	PDR ± DME (n = 105) [total No.]	NPDR + DME (n = 52) [total No.]	No DR (n = 53) [total No.]	No diabetes (n = 54) [total No.]	*P* value ^a^	*P* value ^b^	*P* value ^c^
Sex														
Female, No. (%)	35 (35.4)	21 (38.2)	34 (48.6)	36 (72.0)	.09	.25	.01	29 (27.6)	20 (38.5)	21 (39.6)	23 (42.6)	.13	.90	.76
Male, No. (%)	64 (64.7)	34 (61.8)	36 (51.4)	14 (28.0)				76 (72.4)	32 (61.5)	32 (60.4)	31 (57.4)			
Age, mean (SD), y	62.7 (8.9)	64.2 (10.3)	66.0 (10.9)	58.2 (11.4)	.04	.35	<.001	57.8 (7.0)	60.9 (8.8)	61.3 (8.4)	54.5 (9.2)	.01	.78	<.001
Diabetes duration, mean (SD), y	20.4 (9.4) [99]	18.8 (9.0) [55]	13.9 (7.2) [69]	NA	<.001	.001	NA	12.6 (6.8) [104]	12.2 (7.2) [52]	11.9 (6.0) [53]	NA	.51	.79	NA
Ethnicity, No. (%)														
South Asian	42 (42.4)	14 (25.5)	8 (11.4)	9 (18.0)	<.001	.11	<.001	105 (100)	52 (100)	53 (100)	54 (100)	NA	NA	NA
Other Asian	5 (5.1)	6 (10.9)	4 (5.7)	5 (10.0)				NA	NA	NA	NA	NA	NA	NA
Black	22 (22.2)	16 (29.1)	21 (30.0)	1 (2.0)				NA	NA	NA	NA	NA	NA	NA
White	28 (28.3)	18 (32.7)	32 (45.7)	34 (68.0)				NA	NA	NA	NA	NA	NA	NA
Other^d^	2 (2.0)	1 (1.8)	5 (7.1)	1 (2.0)				NA	NA	NA	NA	NA	NA	NA
Systolic BP, mean (SD), mm Hg	142.9 (20.1) [96]	141.2 (18.9) [50]	140.0 (19.9) [70]	135.1 (19.5) [50]	.35	.73	.19	138.2 (18.3) [105]	140.7 (18.0) [51]	134.5 (18.6) [50]	133.0 (20.6) [41]	.25	.09	.71
Diastolic BP, mean (SD), mm Hg	78.5 (9.5) [96]	77.0 (10.1) [50]	81.0 (11.3) [70]	79.9 (8.4) [50]	.12	.05	.56	78.3 (8.2) [105]	79.4 (8.7) [51]	76.1 (8.5) [50]	82.8 (12.0) [41]	.14	.06	.003
Insulin use, No./total No. (%)														
No	39/99 (39.4)	21/55 (38.2)	47/70 (67.1)	NA	<.001	.001	NA	66/89 (74.2)	34/44 (77.3)	39/45 (86.7)	NA	.10	.25	NA
Yes	60/99 (60.6)	34/55 (61.8)	23/70 (32.9)					23/89 (25.8)	10/44 (22.7)	6/45 (13.3)				
eGFR, mL/min/1.73m^2^, No. (%)														
≥90	24/92 (26.1)	9/50 (18.0)	30/70 (42.9)	19/49 (38.8)	.01	.04	.50	28 (26.7)	10 (19.2)	23 (43.4)	27 (50.0)	.16	.05	.38
60-89	34/92 (37.0)	27/50 (54.0)	30/70 (42.9)	28/49 (57.1)				49 (46.7)	30 (57.7)	24 (45.3)	26 (48.2)			
45-59	11/92 (12.0)	9/50 (18.0)	6/70 (8.6)	2/49 (4.1)				12 (11.4)	7 (13.5)	4 (7.6)	1 (1.9)			
30-44	13/92 (14.1)	2/50 (4.0)	1/70 (1.4)	0				3 (2.9)	4 (7.7)	1 (1.9)	0			
15-29	7/92 (7.6)	1/50 (2.0)	2/70 (2.9)	0				9 (8.6)	1 (1.9)	1 (1.9)	0			
<15	3/92 (3.3)	2/50 (4.0)	1/70 (1.4)	0				4 (3.8)	0	0	0			
Serum creatinine, median (IQR), mg/dL	93.5 (72.0-136) [92]	84.5 (70.0-114.0) [50]	75.5 (64.0-86.0) [70]	70.0 (58.0-79.0) [49]	<.001	.01	.05	84.0 (70.7-106.1)	79.6 (70.7-102.1)	70.7 (61.9-83.0)	70.7 (61.9-79.6)	<.001	.004	.22
HbA_1c_, mean (SD), mmol/mol	67.9 (19.4) [93]	68.9 (19.6) [53]	61.8 (18.5) [70]	38.0 (5.3) [48]	.05	.04	<.001	83.2 (24.2) [105]	76.7 (23.5) [52]	74.3 (26.9) [53]	37.5 (4.1) [53]	.04	.62	<.001
CRP, median (IQR), mg/dL	1.6 (0.7-3.0) [94]	1.1 (0.7-4.0) [54]	1.8 (0.7-3.6) [70]	2.0 (0.9-3.0) [46]	.56	.61	.66	3.0 (1.5-6.5) [92]	2.0 (1.5-4.5) [41]	2.5 (1.6-3.6) [48]	3.0 (2.1-5.4) [48]	.40	.45	.12
HDL, mean (SD), mg/dL	1.2 (0.4) [94]	1.2 (0.4) [54]	1.3 (0.4) [69]	1.6 (0.4) [49]	.56	.68	<.001	1.0 (0.2)	1.1 (0.3)	1.1 (0.2)	1.1 (0.3)	.91	.10	.11
LDL, mean (SD), mg/dL	1.9 (0.9) [90]	1.9 (0.8) [54]	1.9 (0.9) [68]	3.5 (1.3) [49]	.96	.63	<.001	2.6 (0.9)	2.4 (0.9)	2.7 (1.0)	3.1 (0.9)	.81	.18	.02
Triglycerides, median (IQR), mg/dL	1.5 (1.0-2.1) [94]	1.5 (1.1-2.3) [54]	1.5 (1.1-2.4) [70]	1.3 (1.0-2.4) [49]	.64	.81	.42	1.6 (1.3-2.6)	1.8 (1.3-2.5)	2.1 (1.7-2.9)	1.6 (1.3-2.3)	.02	.02	.002
Afamin, mean (SD), μg/mL	22.1 (9.0)	22.2 (9.9)	25.5 (9.4)	23.7 (6.7)	.02	.06	.24	33.0 (11.7)	32.7 (10.9)	37.4 (15.2)	33.0 (13.3)	.05	.08	.12
F2, mean (SD), ng/mL	744.6 (264.4)	665.7 (220.1)	715.1 (208.9)	732.9 (223.4)	.44	.20	.65	1093.7 (412.1) [105]	1019.8 (276.6) [52]	1086.4 (272.3) [53]	1231.7 (437.2) [53]	.91	.22	.04
CFB, mean (SD), μg/mL	506.8 (169.8)	541.6 (153.4)	557.6 (216.6)	444.0 (131.0)	.09	.64	.001	309.3 (83.9) [105]	291.9 (70.7) [52]	338.6 (87.9) [53]	322.2 (85.4) [53]	.04	.003	.33
CFH, mean (SD), μg/mL	502.8 (232.4)	542.9 (258.1)	533.8 (205.5)	482.6 (191.0)	.37	.83	.17	394.6 (110.3) [105]	383.7 (93.2) [52]	380.8 (91.0) [53]	362.4 (82.1) [53]	.43	.87	.28
SerpinA4, mean (SD), μg/mL	10.8 (3.6)	11.2 (5.2)	11.4 (3.7)	11.8 (3.6)	.35	.87	.49	8.4 (2.4) [105]	9.2 (2.5) [52]	9.3 (2.3) [53]	9.4 (2.1) [52]	.03	.87	.80
A2m, mean (SD), mg/mL	4.1 (2.6-7.9)	3.8 (2.1-7.1)	4.3 (2.7-7.9)	3.4 (1.9-5.2)	.89	.46	.10	2.5 (1.9-3.3) [105]	2.8 (2.1-3.6) [52]	2.4 (1.8-3.2) [53]	2.1 (1.7-2.6) [53]	.47	.05	.10
Cystatin C, mean (SD), μg/mL	1.8 (1.2-2.3)	1.7 (1.2-2.5)	1.4 (0.9-1.8)	1.2 (1.0-1.6)	<.001	.01	.38	1.7 (1.4-2.2)	1.6 (1.3-2.0)	1.3 (1.1-1.5)	1.3 (1.1-1.5)	<.001	.003	.98
Lp-PLA2, mean (SD), ng/mL	114.2 (67.4)	113.2 (70.2)	127.4 (65.4)	155.2 (76.7)	.21	.24	.03	159.1 (87.5) [104]	151.6 (75.8) [52]	182.5 (101.5) [53]	202.6 (79.1) [53]	.13	.08	.26
LRG-1, median (IQR), μg/mL	30.5 (20.3-53.1)	30.9 (14.6-56.6)	20.0 (14.2-39.1)	24.5 (16.1-51.8)	<.001	.01	.09	129.7 (99.5-192.3) [105]	113.7 (72.8-178.0) [52]	106.0 (75.1-160.1) [53]	109.4 (90.9-141.0) [53]	.01	.46	.62
8-OHDG, median (IQR), ng/mL	29.8 (22.3-34.2)	28.6 (22.3-33.1)	30.5 (23.2-34.7)	30.9 (21.9-36.1)	.61	.44	.89	24.9 (17.9-32.5) [105]	27.8 (21.3-39.3) [52]	26.2 (19.6-38.6) [53]	24.3 (19.2-29.9) [53]	.23	.51	.23
ApoA1, median (IQR), μg/mL	1971.8 (1451.1-2485.9)	2101.3 (1387.0-2732.1)	2284.2 (1660.7-2910.4)	2526.4 (1781.6-3020.6)	.03	.26	.30	457.1 (369.0-682.3) [105]	507.0 (344.3-653.5) [52]	517.4 (372.7-616.5) [53]	505.4 (421.3-780.5) [53]	.64	.98	.48
ApoC3, median (IQR), μg/mL	92.3 (61.0-123.4)	99.9 (68.4-128.5)	102.2 (74.0-142.3)	97.0 (73.9-125.2)	.09	.80	.69	105.6 (81.9-152.4) [105]	95.9 (67.3-145.0) [52]	121.8 (79.0-155.1) [53]	89.3 (64.4-127.2) [53]	.44	.10	.01
ApoB, median (IQR), μg/mL	501.2 (399.4-639.7)	527.6 (366.1-683.8)	503.4 (366.1-663.1)	693.0 (547.0-880.1)	.88	.74	<.001	578.1 (427.0-765.0) [105]	616.0 (410.0-867.0) [52]	594.0 (441.0-839.0) [53]	700.3 (546.3-885.9) [53]	.52	.98	.44
ApoB/A1, ratio	0.2 (0.2-0.4)	0.2 (0.2-0.3)	0.2 (0.1-0.4)	0.2 (0.2-0.4)	.18	.36	.04	1.2 (1.0-1.5) [105]	1.2 (1.0-1.6) [52]	1.2 (1.0-1.6) [53]	1.1 (0.9-1.5) [53]	.68	.97	.40

Abbreviations: ApoA1, apolipoprotein A1; ApoB, apolipoprotein B; ApoC3, Apolipoprotein C3; A2m, α2 macroglobulin; CFB, complement factor B; CFH, complement factor H; CRP, C-reactive protein; DME, diabetic macular edema; eGFR, estimated glomerular filtration rate; F2, thrombin; HbA_1c_, hemoglobin A_1c_; HDL, high-density lipoprotein; LDL, low-density lipoprotein; Lp-PLA2, lipoprotein-associated phospholipase A2; LRG-1, leucine-rich α2 glycoprotein 1; NA, not applicable; NPDR, nonproliferative diabetic retinopathy; PDR, proliferative diabetic retinopathy; 8-OHDG, 8-hydroxy-2′-deoxyguanosine. SI conversion factor: To convert serum creatinine to μmol/L, multiply by 88.4; to convert serum CRP to mg/L, multiply by 10; to convert serum HDL cholesterol to mmol/L, multiply by 0.0259; to convert serum LDL cholesterol to mmol/L, multiply by 0.0259; to convert serum triglycerides to mmol/L, multiply by 0.0113.

^a^ *P* value for no DR vs PDR.

^b^ *P *value for no DR vs NPDR + DME.

^c^ *P *value for no diabetes vs no DR. For continuous variables, nonparametric Mann-Whitney *U* test used for skewed variables (presented as median [IQR]) and *t* test used for variables approximately normally distributed (presented as mean [SD]). For categorical variables χ^2^ test used or Fisher exact approximation when more than 20% cells have expected frequencies less than 5.

^d^ Other category includes any individual ethnicity that is not named in the UK 2011 Census.

**Figure 1.**
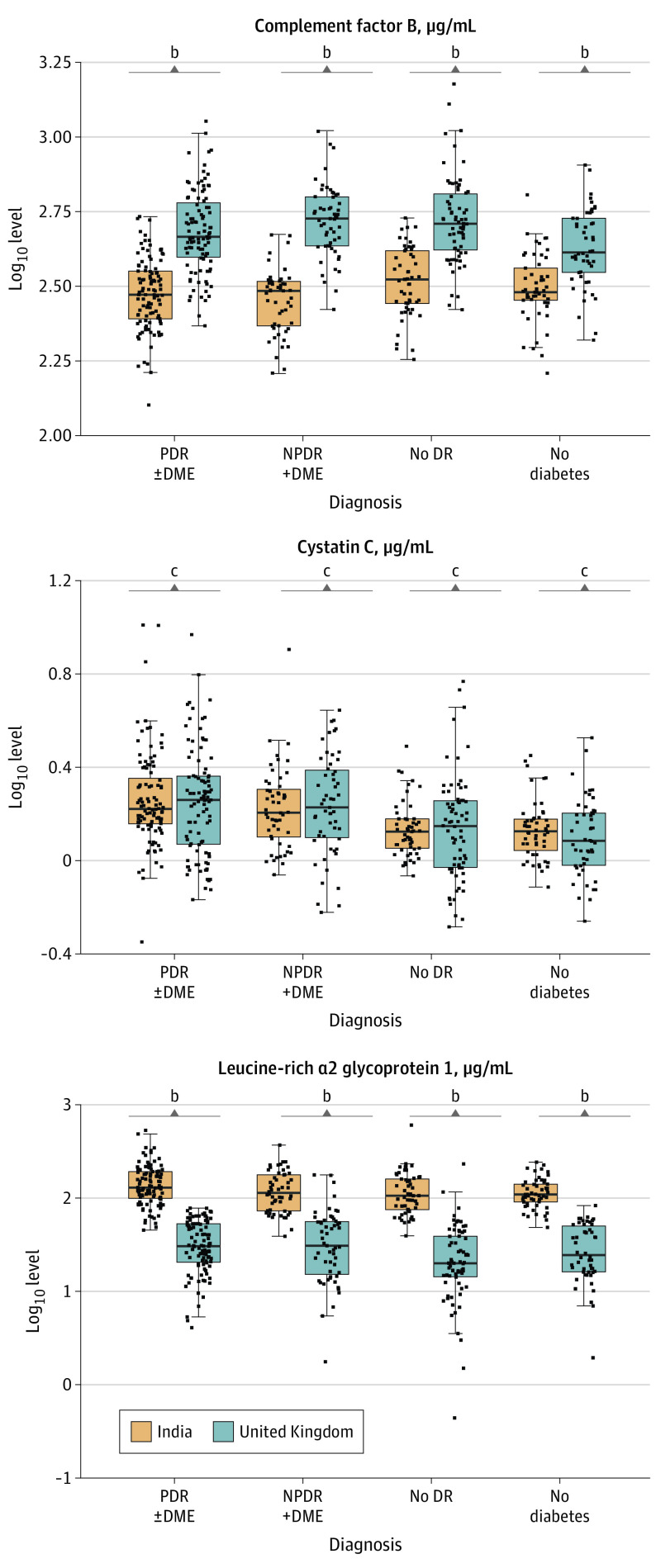
Box Plots Showing Distributions of Biomarkers in the United Kingdom and India^a^ DME indicates diabetic macular edema; NPDR, nonproliferative diabetic retinopathy; PDR, proliferative diabetic retinopathy. ^a^*P* values were generated from the Mann-Whitney *U* (Wilcoxon rank sum) test comparing biomarkers in the United Kingdom and samples from India. ^b^*P* ≤ .0001. ^c^*P* >.05.

The demographic, clinical, and biomarker data of the UK and India cohort, by outcome group, are shown in Table 1, and weighted descriptive analyses comparing No DR and STDR groups used for modeling are shown in eTable 2 in the Supplement. There were significant differences in biomarker values between the UK and India across nearly all disease groups (Figure 1 and eFigure 2 in the Supplement); therefore, we continued as planned to analyze the markers by country as allowed in the sample size calculation but did not further pool the data. Box plots summarizing biomarker values by ethnic group for the UK are presented in eFigure 3 in the Supplement.

### Analysis of Expression Patterns: UK

Adjusted logistic regression results for demographic, clinical, and biomarker data are shown in Table 2. In the adjusted analysis, the following biomarkers were found to be elevated in STDR (groups 3 and 4) relative to the group with no DR (group 2) in the UK: cystatin-C (OR, 1.12 μg/mL; 95% CI, 1.02-1.23 μg/mL; *P* = .02) and LRG-1 (OR, 1.06 μg/mL; 95% CI, 1.00-1.11 μg/mL; *P* = .03). The base model consisting of variables age, duration, race and ethnicity, and HbA_1c_ contributed an AUC of 0.735 (95% CI, 0.652-0.818). The addition of cystatin C to the base model yielded an AUC of 0.779 (95% CI, 0.700-0.857 μg/mL) and LRG-1 to the base model yielded an AUC of 0.763 (95% CI, 0.684-0.842 μg/mL).

**Table 2.** Demographic and Clinical Data Logistic Regression Results for Detecting STDR Groups Relative to No DR, Adjusting for Age, Duration, Race and Ethnicity (in the UK), and HbA_1c_

Variable, per unit increase or as indicated	UK^a^			India^b^		
	[No./Events] OR (95% CI)	*P* value	AUC (95% CI)	[No./Events] OR (95% CI)	*P* value	AUC (95% CI)
Age, per 10-y increase	[215/146] 0.66 (0.42-1.06)	.08	0.735 (0.652-0.818)	[209/156] 0.87 (0.52-1.44)	.58	0.551 (0.447-0.654)
Duration of diabetes, per 10-y	[215/146] 2.42 (1.46-4.00)	.001	0.735 (0.652-0.818)	[209/156] 1.21 (0.62-2.39)	.57	0.551 (0.447-0.654)
HbA_1c_, per 10-mmol/mol	[215/146] 1.19 (0.98-1.45)	.08	0.735 (0.652-0.818)	[209/156] 1.04 (0.91-1.19)	.58	0.551 (0.447-0.654)
Sex						
Female	1 [Reference]	NA	NA	1 [Reference]	NA	NA
Male	[215/146] 1.72 (0.73-4.04)	.21	0.743 (0.661-0.825)	[209/156] 1.18 (0.54-2.60)	.68	0.544 (0.441-0.648)
Ethnicity						
South Asian	[215/146] 3.80 (1.21-11.94)	.02	0.735 (0.652-0.818)	NA	NA	NA
Black	[215/146] 1.29 (0.44-3.77)	.64		NA	NA	NA
White	1 [Reference]	NA	NA	NA	NA	NA
Other	[215/146] 1.12 (0.28-4.57)	.87	0.735 (0.652-0.818)	NA	NA	NA
Systolic blood pressure, per 10 mm Hg	[207/138] 1.04 (0.83-1.29)	.75	0.739 (0.654-0.824)	[205/155] 1.22 (0.99-1.51)	.07	0.609 (0.506-0.712)
Diastolic blood pressure, per 10 mm Hg	[207/138] 0.84 (0.58-1.22)	.37	0.743 (0.658-0.827)	[205/155] 1.57 (0.97-2.54)	.07	0.603 (0.500-0.707)
HDL, per 1 mg/dL	[213/145] 0.60 (0.20-1.85)	.37	0.752 (0.671-0.833)	[209/156] 2.69 (0.58-12.44)	.21	0.593 (0.490-0.697)
LDL, per 1 mg/dL	[208/141] 1.01 (0.62-1.63)	.98	0.740 (0.657-0.823)	[209/156] 0.75 (0.49-1.13)	.17	0.584 (0.482-0.687)
Triglycerides, per 15% increase in mg/dL	[214/145] 1.01 (0.92-1.11)	.87	0.734 (0.652-0.817)	[209/156] 0.85 (0.75-0.96)	.01	0.649 (0.550-0.748)
CRP, per 10% increase in mg/dL	[214/145] 0.99 (0.95-1.03)	.61	0.733 (0.650-0.816)	[180/132] 0.97 (0.92-1.03)	.33	0.610 (0.498-0.721)
Serum Creatinine, per 10% increase in mg/dL	[205/137] 1.26 (1.13-1.41)	<.001	0.779 (0.700-0.858)	[209/156] 1.35 (1.12-1.63)	.002	0.661 (0.564-0.758)
Insulin use						
No insulin	1 [Reference]	NA	NA	1 [Reference]	NA	NA
Insulin	[215/146] 1.68 (0.61-4.61)	.31	0.743 (0.662-0.825)	[177/132] 2.37 (0.66-8.47)	.19	0.564 (0.453-0.675)
Candidate biomarker data						
CFB, per 100 μg/mL increase	[215/146] 0.97 (0.80-1.18)	.76	0.737 (0.654-0.819)	[209/156] 0.48 (0.29-0.78)	.003	0.665 (0.565-0.764)
CFH, per 100 μg/mL increase	[215/146] 1.13 (0.95-1.34)	.16	0.731 (0.648-0.814)	[209/156] 1.03 (0.68-1.58)	.87	0.554 (0.451-0.657)
Serpin A4, per 1 μg/mL increase	[215/146] 0.98 (0.86-1.12)	.82	0.733 (0.651-0.816)	[209/156] 0.97 (0.82-1.14)	.70	0.563 (0.459-0.667)
A2m, per 15% increase in mg/mL	[215/146] 0.99 (0.91-1.09)	.88	0.737 (0.654-0.819)	[209/156] 1.13 (0.99-1.28)	.07	0.604 (0.503-0.705)
Afamin, per 1 μg/mL increase	[215/146] 0.96 (0.92-1.02)	.19	0.750 (0.668-0.831)	[209/156] 0.97 (0.95-1.00)	.06	0.595 (0.494-0.697)
F2, per 100 ng/mL increase	[215/146] 0.96 (0.77-1.21)	.76	0.734 (0.651-0.817)	[209/156] 0.92 (0.80-1.06)	.26	0.588 (0.486-0.691)
Cystatin C, per 10% in μg/mL	[215/146] 1.12 (1.02-1.23)	.02	0.779 (0.700-0.857)	[208/155] 1.38 (1.16-1.63)	<.001	0.696 (0.602-0.791)
Lp-PLA2, per 100 ng/mL increase	[215/146] 0.88 (0.45-1.74)	.72	0.741 (0.659-0.823)	[208/155] 0.68 (0.45-1.02)	.06	0.609 (0.507-0.710)
LRG-1, per 10% increase in μg/mL	[215/146] 1.06 (1.00-1.11)	.03	0.763 (0.684-0.842)	[209/156] 1.03 (0.96-1.10)	.45	0.582 (0.480-0.685)
8-OHDG, per 30% increase in ng/mL	[215/146] 0.99 (0.71-1.39)	.98	0.735 (0.653-0.818)	[209/156] 1.11 (0.89-1.38)	.38	0.581 (0.478-0.684)
ApoA1, per 10% increase in μg/mL	[215/146] 0.98 (0.91-1.05)	.52	0.741 (0.660-0.823)	[209/156] 0.98 (0.92-1.04)	.51	0.576 (0.474-0.678)
ApoC3, per 10% increase in μg/mL	[215/146] 0.96 (0.89-1.04)	.32	0.740 (0.658-0.822)	[209/156] 0.92 (0.83-1.02)	.13	0.615 (0.514-0.716)
ApoB, per 10% increase in μg/mL	[215/146] 1.04 (0.95-1.16)	.35	0.739 (0.658-0.821)	[209/156] 0.99 (0.92-1.06)	.79	0.567 (0.463-0.670)
ApoB/A1, per 10% increase	[215/146] 1.02 (0.98-1.07)	.36	0.742 (0.660-0.824)	[209/156] 1.02 (0.94-1.09)	.65	0.555 (0.453-0.658)

Abbreviations: ApoA1, apolipoprotein A1; ApoB, apolipoprotein B; ApoC3, Apolipoprotein C3; A2m, α2 macroglobulin; AUC, area under the receiver operating characteristic curve; CRP, C-reactive protein; CFB, complement factor B; CFH, complement factor H; DR, diabetic retinopathy; F2, thrombin; HbA_1c_, hemoglobin A_1c_; HDL, high-density lipoprotein; LDL, low-density lipoprotein; Lp-PLA2, lipoprotein-associated phospholipase A2; LRG-1, leucine-rich α2 glycoprotein 1; NA, not applicable; OR, odds ratio; 8-OHDG, 8-hydroxy-2′-deoxyguanosine; STDR, sight-threatening diabetic retinopathy.

^a^ Following adjustment for age, disease duration, race and ethnicity (merging groups other Asian and other), and HbA_1c_. Results for variables age, duration, race and ethnicity, and HbA_1c_ estimated with just these 4 variables in the model. There were 224 participants with no DR or STDR, and missing data in HbA_1c_, age, duration, race and ethnicity, and the variable under consideration were dropped.

^b^ Following adjustment for age, disease duration and HbA_1c_. Results for variables age, diabetes duration, and HbA_1c_ estimated with these 3 variables in the model. There were 210 participants with no DR or STDR, and missing data in HbA_1c_, age, duration, and the variable under consideration were dropped.

### Analysis of Expression Patterns: India

Biomarkers that were found to be associated with STDR (groups 3 and 4) relative to no DR (group 2) in the adjusted analysis were as follows: CFB (0.48 μg/mL per 100 μg/mL increase; 95% CI, 0.29-0.78; *P* = .003), afamin (0.97 μg/mL per 1 μg/mL increase; 95% CI, 0.95-1.00; *P* = .06), cystatin C (1.38 μg/mL per 10% increase; 95% CI, 1.16-1.63; *P* < .001), and Lp-PLA2 (0.68 μg/mL per 10 ng/mL increase; 95% CI, 0.45-1.02; *P* = .06) (Table 2). Interestingly, triglycerides, CFB, Lp-PLA2, and afamin were downregulated in the STDR groups 3 and 4 compared with the no DR group 2. The base model consisting of variables age, duration, and HbA_1c_ contributed an AUC of 0.551 (95% CI, 0.447-0.654). The addition of cystatin C to the base model contributed an AUC of 0.696 (95% CI, 0.602-0.791) and the base model plus CFB contributed an AUC of 0.665 (95% CI, 0.565-0.764).

### Multicollinearity Between Biomarkers

In those variables that reached significance in adjusted analysis (age, duration, HbA_1c_, cystatin C, and LRG-1), the variance inflation factor ranged from 1.1 to 1.2 in the UK. In India, the variance inflation factor ranged 1.1 to 1.6 for variables age, duration, HbA_1c_, cystatin C, afamin, A2m, Lp-PLA2, and CFB, which were variables considered for inclusion in the multimarker panel, indicating that multicollinearity is unlikely to cause problems for our analysis.

### Combination of Biomarkers to Improve Discriminatory Power

**Figure 2.**
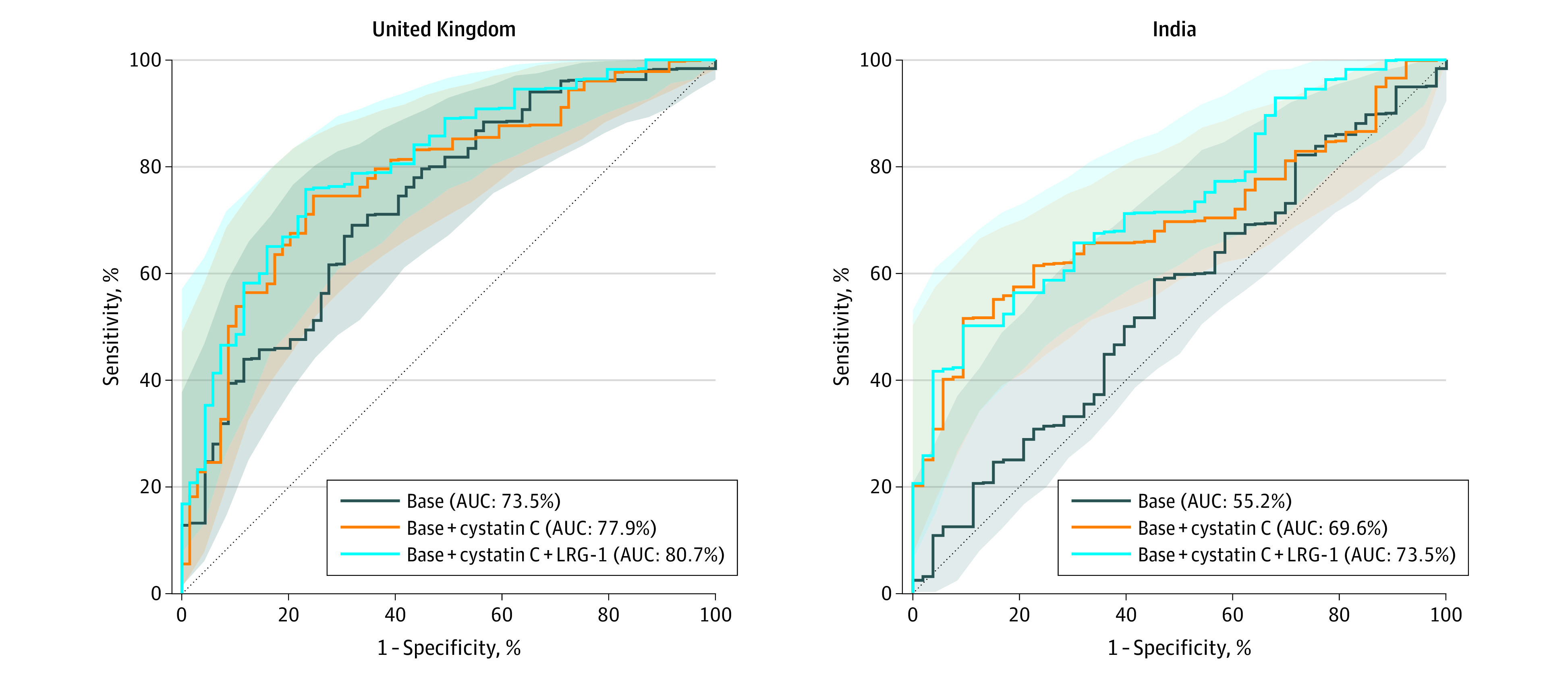
Diagnostic Performance of Combination of Biomarkers Selected From a Forward-Stepwise Routine for the United Kingdom^a^ and India^b^ Area under the receiver operating characteristic curve (AUC) and 95% CIs are presented. LRG-1 indicates leucine-rich α2 glycoprotein 1. ^a^In the United Kingdom, a total of 215 patients with 146 sight-threatening diabetic retinopathy (STDR) events were used to derive the final models. The base model includes age, diabetes duration, race and ethnicity (South Asian, Black, other) and hemoglobin A_1c_ (HbA_1c_). ^b^In India, a total of 208 patients with 155 STDR events were used to derive the final models. The base model includes age, diabetes duration, and HbA_1c_.

For the UK model, the forward routine with selected additional biomarkers LRG-1 and cystatin C together yielded an AUC of 0.807 (95% CI, 0.734-0.879) on top of variables in the base model. In India, additional biomarkers included cystatin C and CFB, with a combined AUC of 0.735 (95% CI, 0.647-0.822) (eTable 2 in the Supplement, Table 3, and Figure 2).

**Table 3.** Assessment of Performance of Final Models for UK and India Populations Based on Varying Thresholds

Strategy^a^	AUC (95% CI)	Metric	Threshold	Sensitivity, %	Specificity, %	Population PPV	Population NPV	LR+	LR−	No of people per 1000 at risk in a prospective population		No of STDR events per 1000 in a prospective population	
										High risk	Low risk	Identified	Not identified
UK (n = 215)													
Screen all	NA	NA	0	100.0	0.0	6.0	0.0	1.0	Infinity^b^	1000	0	60	0
Base model	0.735 (0.652-0.818)	Youden	0.055	69.0	68.1	12.2	97.2	2.2	0.5	340	660	42	18
		80% Sensitivity	0.039	80.1	50.7	9.5	97.5	1.6	0.4	512	488	47	13
		90% Sensitivity	0.027	90.7	36.2	8.4	98.4	1.4	0.3	656	344	56	4
		80% Specificity	0.087	45.9	81.2	13.5	95.9	2.4	0.7	205	795	28	32
		90% Specificity	0.123	39.3	91.3	22.5	95.9	4.5	0.7	107	893	23	37
Base model + cystatin C (model 1)	0.779 (0.700-0.857)	Youden	0.058	74.5	75.4	16.3	97.9	3.0	0.3	274	726	47	13
		80% Sensitivity	0.043	81.2	60.9	11.8	98.1	2.1	0.3	419	581	51	9
		90% Sensitivity	0.022	91.1	29.0	7.6	98.1	1.3	0.3	721	279	56	4
		80% Specificity	0.072	65.3	81.2	18.2	97.3	3.5	0.4	219	781	37	23
		90% Specificity	0.124	50.1	91.3	27.0	96.6	5.8	0.5	112	888	33	27
Base model + cystatin C and LRG-1 (model 2)	0.807 (0.734-0.879)	Youden	0.067	75.7	76.8	17.3	98	3.3	0.3	265	735	47	13
		80% Sensitivity	0.038	80.5	60.9	11.7	98	2.1	0.3	419	581	47	13
		90% Sensitivity	0.022	90.8	44.9	9.6	98.7	1.6	0.2	572	428	56	4
		80% Specificity	0.085	66.8	81.2	18.6	97.4	3.5	0.4	219	781	42	18
		90% Specificity	0.152	46.5	91.3	25.6	96.4	5.3	0.6	112	888	28	32
India (n = 208)													
Screen all	NA	NA	0	100.0	0.0	5.2	0.0	1.0	Infinity^b^	1000	0	52	0
Base model	0.552 (0.448-0.655)	Youden	0.052	58.8	54.7	6.6	96.0	1.3	0.8	462	538	29	23
		80% Sensitivity	0.046	80.2	28.3	5.8	96.3	1.1	0.7	721	279	43	9
		90% Sensitivity	0.040	91.6	9.4	5.3	95.4	1.0	0.9	904	96	48	4
		80% Specificity	0.061	24.9	81.1	6.8	95.2	1.3	0.9	192	808	14	38
		90% Specificity	0.066	12.4	90.6	6.7	95.0	1.3	1.0	96	904	5	47
Base model + cystatin C (model 1)	0.696 (0.602-0.791)	Youden	0.086	51.5	90.6	23	97.1	5.5	0.5	116	884	27	25
		80% Sensitivity	0.023	81.1	30.2	6.0	96.7	1.2	0.6	704	296	42	10
		90% Sensitivity	0.013	91.5	13.2	5.5	96.6	1.1	0.6	870	130	48	4
		80% Specificity	0.062	57.4	81.1	14.3	97.2	3.0	0.5	209	791	30	22
		90% Specificity	0.086	51.5	90.6	23	97.1	5.5	0.5	116	884	27	25
Base model + cystatin C and CFB (model 2)	0.735 (0.647-0.822)	Youden	0.093	50.1	90.6	22.6	97.1	5.3	0.6	115	885	26	26
		80% Sensitivity	0.024	80.8	35.8	6.5	97.1	1.3	0.5	650	350	42	10
		90% Sensitivity	0.015	91.2	32.1	6.9	98.5	1.3	0.3	691	309	47	5
		80% Specificity	0.071	56.3	81.1	14.1	97.1	3.0	0.5	208	792	29	23
		90% Specificity	0.093	50.1	90.6	22.6	97.1	5.3	0.6	115	885	26	26

Abbreviations: CFB, complement factor B; LRG-1, leucine-rich α2 glycoprotein 1; LR+, positive likelihood ratio; LR−, negative likelihood ratio; NA, not applicable; NPV, negative predictive value; PPV, positive predictive value; STDR, sight-threatening diabetic retinopathy.

^a^ The base models include age, duration, race and ethnicity (in the UK), and hemoglobin A_1c_. In the UK, model 1 includes the base model and cystatin C; model 2 includes the base model with both cystatin C and LRG-1. In India, model 1 includes the base model and cystatin C; model 2 includes the base model with both cystatin C and CFB. eTable 3 in the Supplement contains the multivariable logistic regression results for models 1 and 2.

^b^ LR− is infinity when screening all patients, as test specificity is 0.

### Diagnostic Test Accuracy and Applications in a Prospective UK and India Population

STDR detection relative to no DR in 1000 individuals in the UK and India populations using the biomarker prescreening strategy is shown in Table 3. In a UK population with estimated prevalence of STDR relative to no DR of 6%, if 1000 people with no DR or STDR undergo retinal screening, 60 out of 1000 people will be identified as having STDR. If the model including cystatin C without LRG-1 is used to prescreen these 1000 patients, 274 patients would be classified as high risk of STDR, and 47 out of 60 patients with STDR will be detected with a test that maximizes the Youden index. The remaining 13 patients with STDR would need to be identified as per current practice.

In India, retinal screening on 1000 people with no DR or STDR will identify 52 individuals with STDR based on the prevalence of STDR relative to no DR of 5.2%. If cystatin C was used to test these patients first, a total of 115 out of 1000 will be triaged for retinal screening and 29 out of 52 STDR will be identified, using a targeted threshold of more than 8.6%. At 80% sensitivity (30% specificity), we would need to screen 704 patients out of 1000 to detect 42 out of 52 STDR cases, with approximately 97% of low-risk patients correctly identified as having no STDR.

### Sensitivity Analysis

As both cystatin C and serum creatinine had comparable AUC, cystatin C (log) was substituted with serum creatinine (log). In the UK, only 205 patients with 137 STDR events had available data to use serum creatinine instead of cystatin C in the models. The base model with serum creatinine instead of cystatin C, yielded an AUC of 0.779, and when combining with LRG-1, the AUC was 0.791 (eFigure 4 in the Supplement). Using the same sample of 205 patients yielded an AUC of 0.799 in the model using cystatin C instead of serum creatinine. In India, replacing cystatin C with serum creatinine reduced the AUC to 0.661 in a model with the known risk factors and serum creatinine, and combined with CFB, the AUC was 0.731 compared with 0.735 in a model with cystatin C and CFB. As cystatin C is also a marker of kidney disease, we conducted a subgroup analysis in patients with normal kidney function, ie, eGFR of 90 mL/min/1.73m^2^ or greater (63 in the UK [33 STDR events] and 61 in India [38 STDR events]). In patients with an eGFR of 90 mL/min/1.73m^2 ^or greater, the presence of STDR correlated with elevated cystatin C levels in the UK (median, 1.1; IQR, 0.74-1.5 vs median, 1.7; IQR, 1.0-1.9; weighted Mann-Whitney *U* test, *P* = .004; age- and duration-adjusted weighted logistic regression, *P* = .05) and similarly in India (median, 1.1; IQR, 1.1-1.3 vs median, 1.4; IQR, 1.1-1.5; weighted Mann-Whitney *U* test, *P* = .21; age- and duration-adjusted weighted logistic regression, *P* = .04).

## Discussion

This cross-sectional study evaluated previously verified circulating biomarkers for DR to establish if these markers could identify STDR from no DR in clinical practice. Many of the chosen markers did not perform as well as previously reported.^11,12^ However, our results suggest possible use of circulating cystatin C as a triage test, as well as the known risk factors of age, race and ethnicity, duration of diabetes, and HbA_1c_^13,14^ as a potentially valid method to identify individuals with STDR.

In the UK cohort, only cystatin C and LRG-1 showed significant differences between no DR (group 2) and STDR (groups 3 and 4). Comparing the STDR and no DR groups in India, CFB, A2m, afamin, cystatin C, and Lp-PLA2 were significantly different at the α = 1% threshold. Owing to interlaboratory variations in some of the biomarkers investigated, we opted to include only the most stable biomarker in the strategy with near-acceptable discrimination in both study cohorts.

When cystatin C levels were added to age, duration of diabetes, race and ethnicity, and HbA_1c_ in the UK, the analysis showed acceptable performance, with an AUC of 0.779 compared with an AUC of 0.735 without cystatin C. In India, the model with age, duration, and HbA_1c_ had performance closer to chance, with an AUC of 0.552, and adding cystatin C had close to acceptable performance with an AUC of 0.696. The validity of this strategy was similar in the UK and India; therefore, results suggest that this biomarker was useful in both countries.^15^

Both models achieved adequate sensitivity while preserving test specificity to an extent; however, the UK models had higher specificity across all tested thresholds. This might be partly attributable to the duration of diabetes not being as accurate in detecting STDR in India unlike the UK, where this was one of the strongest indicators of STDR. Nonetheless, our results show stability across both data sets. Several studies done globally have demonstrated an association between cystatin C and DR.^16,17,18,19,20^

### Strengths and Limitations

This study had several strengths. First, both the UK and India models achieved close to acceptable discrimination. Second, the models relied mostly on routinely collected data, except for cystatin C. However, a point-of-care cystatin C biosensor is feasible to produce, will not require skilled workers to perform, and may be accessible as a web-based application for self-monitoring by patients.^21^ Third, in contrast to many other published studies, which often use mass spectrometry of plasma,^4,11,12^ we opted for ELISA analyses of serum to remain close to what is routinely available in a health care setting.^22^ Fourth, data for cystatin C, used in our final model, did reflect that seen in the literature.^18,19^

However, there were several limitations. First, we were unable to pool the samples from UK and India owing to differences in the distribution of some biomarkers. More studies are required to understand intercountry variations in the profiles of these biomarkers. Second, further validation in a cohort representative of the real-world prevalence of STDR and not oversampled for cases is needed. Third, as these markers were analyzed in stored and freeze-thawed serum only, further validation of our prescreening strategy should be undertaken in a community screening setting using finger-prick blood test. Notably, similar low-cost point-of-care HbA_1c_ kits are already available for community screening.^23^ Fourth, validation of a cystatin C point-of-care kit is needed before use in a health care– or community-screening setting. Fifth, patients with NPDR but without DME were not recruited into this study, and therefore, statistical models may not generalize to all patients with diabetes or represent population-level risk of STDR. Sixth, serum cystatin C has also been shown to be an early marker of diabetic kidney disease,^24^ but our results showed that cystatin C remained elevated in STDR in a subgroup of patients with normal eGFR.

## Conclusions

Results of this cross-sectional study suggest that serum cystatin C had good discrimination power in the UK and India. Although retinal imaging is the criterion standard for DR screening and should be advocated for all patients with diabetes on an annual or biennial basis, this is not always feasible in low- and middle-income countries. In such low-resource settings, prescreening models with cystatin-C may potentially be used to identify those who need prioritization for retinal screening.

## Notes

Supplement. **eFigure 1.** Flowchart **eFigure 2.** Box Plots Showing Distributions of Biomarkers in UK vs India^a^ **eFigure 3.** Box Plots by Ethnic Groups in the UK **eTable 1.** Suppliers of ELISA Kits for Biomarker Analysis, Limits of Detection, and Sample Dilutions **eTable 2.** Weighted^a^ Summary Demographic, Clinical, and Biomarker Data by Outcome Group (STDR vs no DR) and Country **eTable 3.** Multivariable Logistic Regression Results (Final Models) for STDR vs no DR in UK and India **eFigure 4.** Sensitivity Analysis—ROC Curves Showing Discriminatory Ability With Serum Creatinine Instead of Cystatin C for UK^a^ and India^b ^
eReferences
