# Supplementary material for: Multicenter Evaluation of Diagnostic Circulating Biomarkers to Detect Sight-Threatening Diabetic Retinopathy
Source: JAMA Ophthalmol. 2022 May 5;140(6):587–97. doi: 10.1001/jamaophthalmol.2022.1175 (PMC9073659; doi:10.1001/jamaophthalmol.2022.1175)
Supplement: Supplement. — eFigure 1. Flowchart eFigure 2. Box Plots Showing Distributions of Biomarkers in UK vs Indiaa eFigure 3. Box Plots by Ethnic Groups in the UK eTable 1. Suppliers of ELISA Kits for Biomarker Analysis, Limits of Detection, and Sample Dilutions eTable 2. Weighteda Summary Demographic, Clinical, and Biomarker Data by Outcome Group (STDR vs no DR) and Country eTable 3. Multivariable Logistic Regression Results (Final Models) for STDR vs no DR in UK and India eFigure 4. Sensitivity Analysis—ROC Curves Showing Discriminatory Ability With Serum Creatinine Instead of Cystatin C for UKa and Indiab eReferences [file jamaophthalmol-e221175-s001.pdf]

Supplementary Online Content

Gurudas S, Frudd K, Maheshwari JJ, et al. Multicenter evaluation of diagnostic circulating biomarkers to detect sight-threatening diabetic retinopathy. *JAMA Ophthalmol*. Published online May 5, 2022. doi:10.1001/jamaophthalmol.2022.1175

eFigure 1. Flowchart

eFigure 2. Box Plots Showing Distributions of Biomarkers in UK vs India[a]

eFigure 3. Box Plots by Ethnic Groups in the UK

eTable 1. Suppliers of ELISA Kits for Biomarker Analysis, Limits of Detection, and Sample Dilutions

eTable 2. Weighted[a] Summary Demographic, Clinical, and Biomarker Data by Outcome Group (STDR vs no DR) and Country

eTable 3. Multivariable Logistic Regression Results (Final Models) for STDR vs no DR in UK and India

eFigure 4. Sensitivity Analysis—ROC Curves Showing Discriminatory Ability With Serum Creatinine Instead of Cystatin C for UK[a] and India[b]

eReferences

This supplementary material has been provided by the authors to give readers additional information about their work.

© 2022 Gurudas S et al. *JAMA Ophthalmology.*

eFigure 1. Flowchart

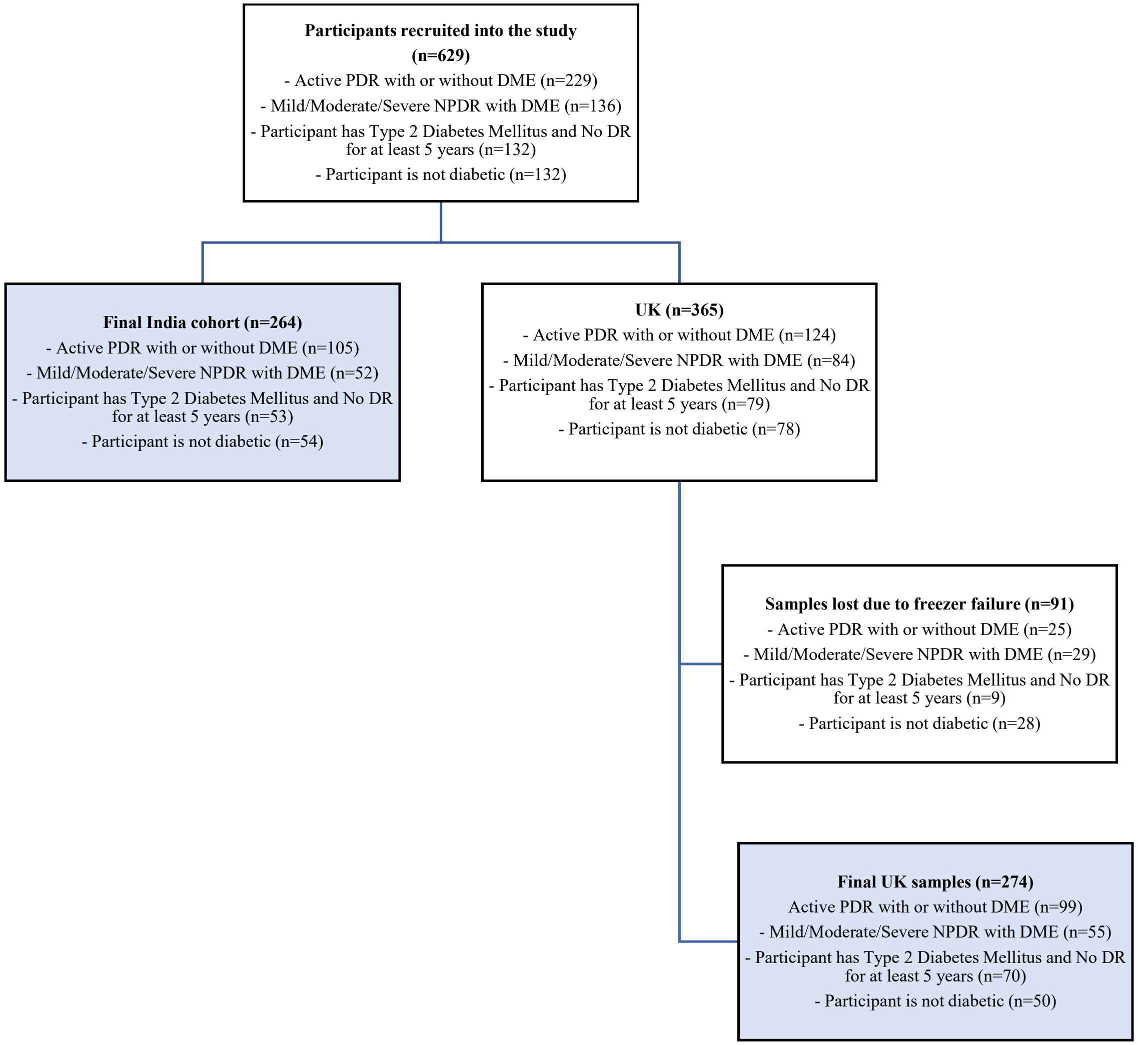

© 2022 Gurudas S et al. *JAMA Ophthalmology.*

Abbreviations: DMO-diabetic macular oedema; NPDR- non-proliferative diabetic retinopathy; PDR-proliferative diabetic retinopathy; UK-United Kingdom.

© 2022 Gurudas S et al. *JAMA Ophthalmology.*

eFigure 2. Box Plots Showing Distributions of Biomarkers in UK vs India[a]

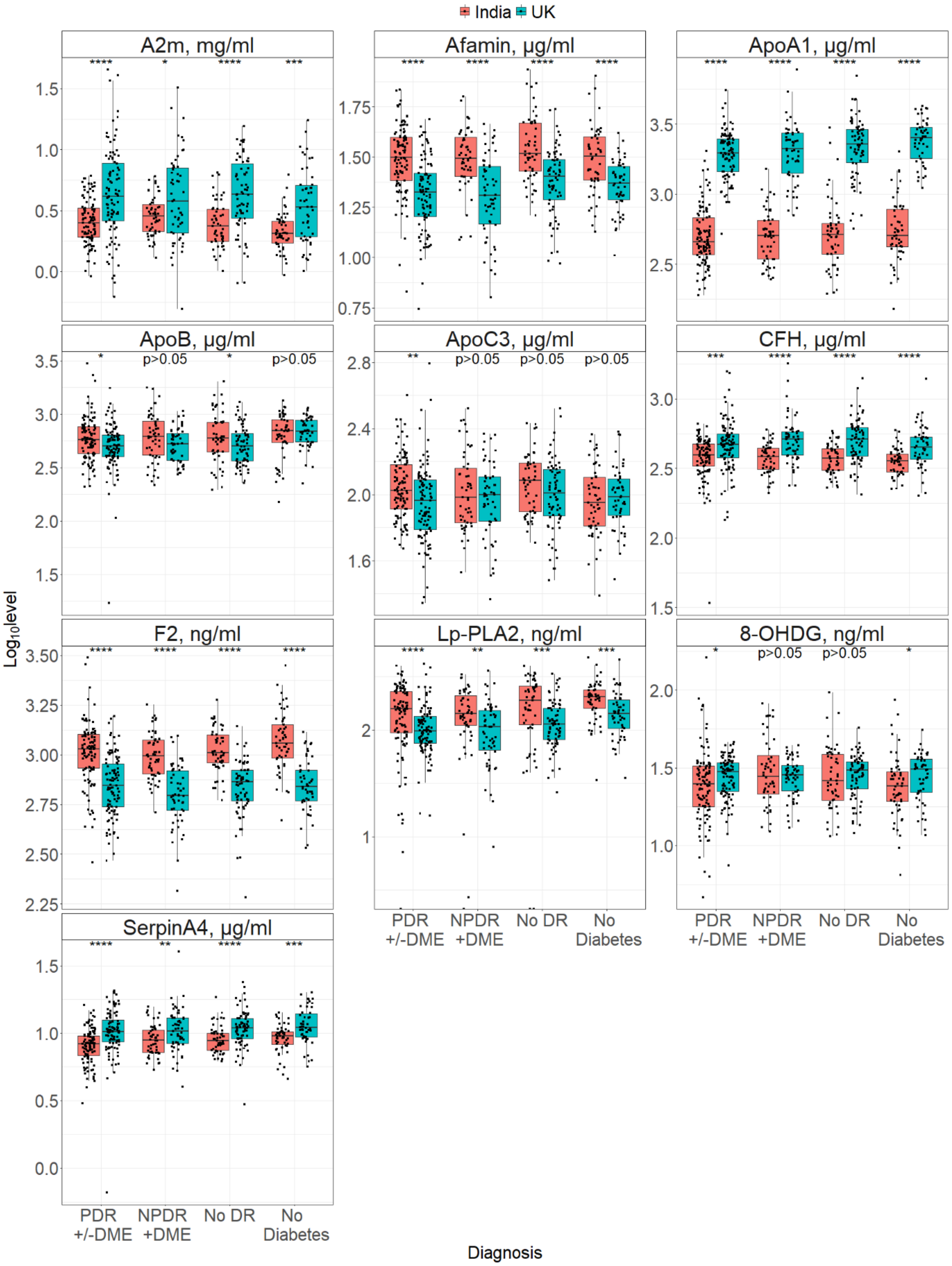

Abbreviations: CFH- Complement factor H, A2m- Alpha-2 macroglobulin, F2-Thrombin, 8-OHDG- 8-hydroxy-2' -deoxyguanosine, APOA1- Apolipoprotein A1, APOC3- Apolipoprotein C3, APOB- Apolipoprotein B, DME-diabetic macular oedema; NPDR- non-proliferative diabetic retinopathy; PDR-proliferative diabetic retinopathy; UK-United Kingdom.

© 2022 Gurudas S et al. *JAMA Ophthalmology.*

[a] P-values were generated from the Mann-Whitney (Wilcoxon rank-sum) test comparing biomarkers in UK and India samples and the following conventions were used to indicate statistical significance: ****, $p \leq .0001$. ; ***, $p \leq .001$; **, $p \leq .01$; *, $p \leq 0.05$

© 2022 Gurudas S et al. *JAMA Ophthalmology.*

eFigure 3. Box Plots by Ethnic Groups in the UK

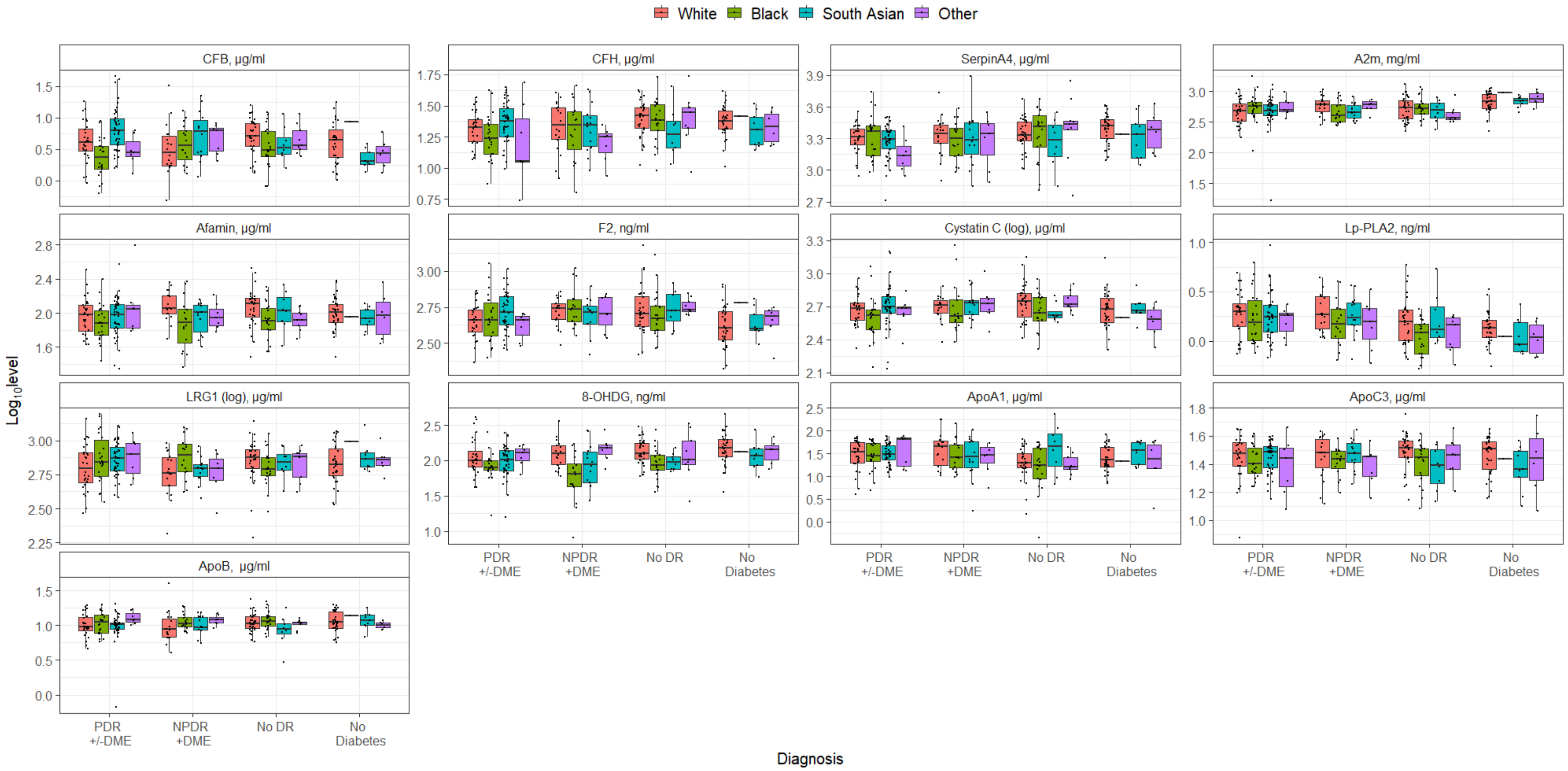

Abbreviations: CRP- C-reactive protein, HDL- High density lipoprotein, LDL- Low density lipoprotein, CFB- Complement factor B, CFH- Complement factor H, A2m- Alpha-2 macroglobulin, F2-Thrombin, Lp-PLA2- Lipoprotein-associated Phospholipase A2, LRG1- Leucine Rich Alpha-2-Glycoprotein 1, 8-OHDG- 8-hydroxy-2' -deoxyguanosine, ApoA1- Apolipoprotein A1, ApoC3- Apolipoprotein C3, ApoB- Apolipoprotein B, DME-diabetic macular oedema; NPDR- non-proliferative diabetic retinopathy; PDR-proliferative diabetic retinopathy

© 2022 Gurudas S et al. *JAMA Ophthalmology.*

eTable 1. Suppliers of ELISA Kits for Biomarker Analysis, Limits of Detection, and Sample Dilutions

Marker protein	ELISA supplier	Cat Code	Type of ELISA	Kit Detection range	Serum dilution used
CFB	Abcam	ab137973	Sandwich ELISA	4.375-140 ng/ml	1:20 000
CFH	R&D DuoSet	DY4779-15	Sandwich ELISA	0.206-150 ng/ml	1:70 000
Serpin A4	R&D DuoSet	DY1669	Sandwich ELISA	125-8000 pg/ml	1:7000
Apo A1	R&D DuoSet	DY3664-05	Sandwich ELISA	3.13-200 ng/ml	1:7000 1:35 000 1:70 000
Apo B	R&D	DAPB00	Sandwich ELISA	39.1 - 2,500 ng/ml	1:1000
Apo C3	Abcam	ab154131	Sandwich ELISA	0 – 0.5 µg/ml	1:4000
A2M	R&D DuoSet	DY1938	Sandwich ELISA	0.625-40 ng/ml	1:700 000
Cystatin C	R&D DuoSet	DY1196	Sandwich ELISA	62.5 – 2000 pg/ml	1:2000
Thrombin F2	Abcam	ab108909	Sandwich ELISA	0.313 - 20 ng/ml	1:250
Afamin	R&D DuoSet	DY8065-05	Sandwich ELISA	78.1 - 5000 pg/ml	1:20 000
LRG1	IBL	#27769	Sandwich ELISA	1.56 ～ 100 ng/mL	1:2000
Lp-PLA2	R&D	DPLG70	Sandwich ELISA	0.8 - 50 ng/mL	1:10
8-OHDG	Abcam	ab201734	Competitive ELISA	0.94 - 60 ng/ml	1:10

Abbreviations: CFB- Complement factor B, CFH- Complement factor H, A2m- Alpha-2 macroglobulin, F2-Thrombin, Lp-PLA2- Lipoprotein-associated Phospholipase A2, LRG1- Leucine Rich Alpha-2-Glycoprotein 1, 8-OHDG- 8-hydroxy-2' -deoxyguanosine, ApoA1- Apolipoprotein A1, ApoC3- Apolipoprotein C3, ApoB-Apolipoprotein B, DMO-diabetic macular oedema; NPDR- non-proliferative diabetic retinopathy; PDR-proliferative diabetic retinopathy; UK-United Kingdom.

© 2022 Gurudas S et al. *JAMA Ophthalmology.*

eTable 2. Weighted[a] Summary Demographic, Clinical, and Biomarker Data by Outcome Group (STDR vs no DR) and Country

	UK				India			
Variable mean (SE), median (q1-q3) or %	N	No DR (N=70)	Weighted STDR (N=154)	P-value[b]	N	No DR (N=53)	Weighted STDR (N=157)	P-value[b]
**Demographic and clinical data**								
**Female**<br>**Male**	224	48.6%<br>51.4%	37.8%<br>62.2%	.20	210	39.6%<br>60.4%	36.9%<br>63.1%	.76
**Age (years)**	224	66.0(SE 1.3)	64.0(SE 1.2)	.27	210	61.3(1.1)	60.4(1.0)	.55
**Diabetes duration (years)**	223	13.9(SE 0.9)	19.0(SE 1.1)	<.001	209	11.9(SE 0.8)	12.3(SE 0.9)	.74
**Ethnicity**<br>**White**<br>**Black**<br>**South Asian**<br>**Other Asian**<br>**Other**	224	 45.7%<br>30.0%<br>11.4%<br>5.7%<br>7.1%	 32.2%<br>28.2%<br>27.6%<br>10.2%<br>1.8%	.03	-			
**Systolic BP (mmHg)**	216	140.0(SE 2.4)	141.4(SE 2.3)	.66	206	134.5(SE 2.6)	140.4(SE 2.2)	.09
**Diastolic BP (mmHg)**	216	81.0(SE 1.3)	77.2(SE 1.2)	.04	206	76.1(SE 1.2)	79.2(SE 1.0)	.05
**Insulin use**<br>**No**<br>**Yes**	224	 67.1%<br>32.9%	 38.3%<br>61.7%	<.001	178	 88.1%<br>11.9%	 76.4%<br>23.6%	.14
**eGFR, mL/min/1.73m^2**	212	84.5(SE 3.4)	71.4(SE 3.1)	.005	210	86.6(SE 3.3)	73.4(SE 2.7)	.01
**Serum Creatinine (mg/dl)**	212	75.5(64.0-86.0)	85.0(70.0-115.0)	.002	210	70.7(61.9-83.0)	79.6(70.7-102.5)	.001
**HbA1c (mmol/mol)**	216	61.8(SE 2.2)	68.7(SE 2.3)	.03	210	74.3(SE 3.7)	77.6(SE 2.8)	.47
**CRP (mg/dl)**	218	1.8(0.7-3.6)	1.1(0.7-3.8)	.60	181	2.5(1.6-3.6)	2.0(1.5-4.6)	.56
**HDL (mg/dl)**	217	1.3(SE 0.05)	1.2(SE 0.05)	.65	210	1.1(SE 0.03)	1.1(SE 0.03)	.13
**LDL (mg/dl)**	212	1.9(SE 0.1)	1.9(SE 0.1)	.65	210	2.7(SE 0.1)	2.5(SE 0.1)	.20
**Triglycerides (mg/dl)**	218	1.5(1.1-2.4)	1.5(1.1-2.3)	.78	210	2.1(1.7-2.9)	1.8(1.3- 2.5)	.01
**Biomarker data**								
**Afamin (µg/ml)**	224	25.5(SE 1.1)	22.2(SE 1.2)	.04	210	37.4(SE 2.1)	32.8(SE 1.3)	.06
**F2 (ng/ml)**	224	715.1(SE 24.8)	675.7(SE 26.0)	.28	210	1086.4(SE 37.1)	1030.4(SE 33.1)	.26
**CFB (µg/ml)**	224	557.6(SE 25.8)	537.3(SE 18.1)	.52	210	338.6(SE 12.0)	294.4(SE 8.4)	.003
**CFH (µg/ml)**	224	533.8(SE 24.4)	537.9(SE 30.4)	.92	210	380.8(SE 12.4)	385.2(SE 11.1)	.79
**SerpinA4 (µg/ml)**	224	11.4(SE 0.4)	11.2(SE 0.6)	.81	210	9.3(SE 0.3)	9.1(SE 0.3)	.65
**A2m (mg/ml)**	224	4.3(2.7-7.9)	3.8(2.1-7.1)	.49	210	2.4(1.8-3.2)	2.8(2.1-3.5)	.04
**Cystatin C (µg/ml)**	224	1.4(0.9-1.8)	1.7(1.2-2.5)	.004	210	1.3(1.1-1.5)	1.6(1.3-2.1)	.001
**Lp-PLA2 (ng/ml)**	224	127.4(SE 7.8)	113.3(SE 8.3)	.22	209	182.5(E 13.8)	152.6(SE 9.0)	.07
**LRG1 (µg/ml)**	224	20.0(14.2-39.1)	30.9(15.9-55.4)	.01	210	106.0(75.1-160.1)	117.9(74.6-178.9)	.30
**8-OHDG (ng/ml)**	224	30.5(23.2-34.7)	28.6(22.3-33.1)	.42	210	26.2(19.6-38.6)	27.4(20.6-37.0)	.65
**ApoA1 (µg/ml)**	224	2284.2(1660.7-2910.4)	2101.3(1387.0-2730.2)	.18	210	517.4(372.7-616.5)	503.0(345.0-655.8)	.93
**ApoC3 (µg/ml)**	224	102.2(74.0-142.3)	99.8(68.4-128.5)	.66	210	121.8(79.0-155.1)	96.1(67.4-146.9)	.13
**ApoB (µg/ml)**	224	503.4(366.1-663.1)	527.6(374.8-666.8)	.74	210	594.0(441.0-839.0)	614.6(415.0-850.0)	.95
**ApoB/A1 (ratio)**	224	0.2(0.1-0.4)	0.2(0.2-0.3)	.28	210	1.2(1.0-1.6)	1.2(1.0-1.6)	.93

Abbreviations: CRP- C-reactive protein, HDL- High density lipoprotein, LDL- Low density lipoprotein, eGFR- estimated glomerular filtration rate, CFB- Complement factor B, CFH- Complement factor H, A2m- Alpha-2 macroglobulin, F2-Thrombin, Lp-PLA2- Lipoprotein-associated Phospholipase A2, LRG1- Leucine Rich Alpha-2-

© 2022 Gurudas S et al. *JAMA Ophthalmology.*

Glycoprotein 1, 8-OHDG- 8-hydroxy-2' -deoxyguanosine, ApoA1- Apolipoprotein A1, ApoC3- Apolipoprotein C3, ApoB- Apolipoprotein B, DME-diabetic macular oedema; NPDR- non-proliferative diabetic retinopathy; PDR-proliferative diabetic retinopathy; UK-United Kingdom.

Groups ii) No DR with Type-2 diabetes for at least 5 years and STDR groups iii) and iv) were used for statistical modelling.

[a] Population proportions used to derive weights in the UK given as follows; 93.96% No DR, 5.28% NPDR+DME, 0.76% PDR and for India as follows; 94.80% No DR, 4.45% NPDR+DME, 0.75% PDR. Study sample proportions in N=224 participants in the UK were 31.25% for No DR, 24.55% for NPDR + DME, 44.20% for PDR. Study sample proportions in N=210 participants in India were; 25.24% for No DR, 24.76% for NPDR + DME and 50.00% for PDR.

[b] P-value for No DR vs STDR. For continuous variables, probability weighted Mann Whitney test used for skewed variables (presented as Median [IQR]), generated using the *survey* package[1] in R and probability weighted t-test with linearized standard errors used for variables approximately normally distributed (presented as Mean(SE)) using Stata. For categorical variables probability weighted χ^2 - test was used using Stata.

© 2022 Gurudas S et al. *JAMA Ophthalmology.*

eTable 3. Multivariable Logistic Regression Results (Final Models) for STDR vs no DR in UK and India

	**UK** ***N=215***				**India** ***N=208***			
	**Model 1**		**Model 2**		**Model 1**		**Model 2**	
**Variable**	Odds ratio (95% CI)	P-value	Odds ratio (95% CI)	P-value	Odds ratio (95% CI)	P-value	Odds ratio (95% CI)	P-value
**Age, per 10-year increase**	0.65(0.41-1.06)	.08	0.58(0.35-0.95)	.03	0.50 (0.26-0.95)	.04	0.43(0.21-0.86)	.02
**Duration, per 10-year increase**	2.19(1.31-3.67)	.003	2.48(1.47-4.17)	.001	1.63(0.77-3.45)	.20	1.63(0.77-3.43)	.20
**White**	Ref	-	Ref	-	-	-	-	-
**Black**	1.97(0.63-6.21)	.25	2.45(0.73-8.25)	.15				
**South Asian**	3.47(0.92-13.17)	.07	2.75(0.73-10.39)	.14				
**Other**	1.71(0.40-7.22)	.47	2.29(0.50-10.50)	.29				
**HbA1c, per 10-mmol/mol increase**	1.32(1.08-1.62)	.01	1.29(1.03-1.61)	.03	1.10(0.94-1.29)	.22	1.13(0.96-1.33)	.13
**Cystatin C, per 10% µg /ml increase**	1.12(1.02-1.23)	.02	1.15(1.05-1.25)	.001	1.38(1.16-1.63)	<.001	1.36(1.15-1.62)	<.001
**LRG1, per 10% µg /ml increase**	-	-	1.08(1.01-1.15)	.02	-	-	-	-
**CFB, per 100 µg /ml increase**	-	-	-	-	-	-	0.49(0.28-0.84)	.01

Abbreviations: HbA1c- Glycated Haemoglobin, CFB- Complement factor B, LRG1- Leucine Rich Alpha-2-Glycoprotein 1
Groups ii) No DR with Type-2 diabetes for at least 5 years and STDR groups iii) and iv) were used for statistical modelling. Weighted logistic regression results presented with 95% confidence intervals generated using robust standard errors, population proportions in the UK given as follows; 93.96% No DR, 5.28% NPDR+DME, 0.76% PDR and for India as follows; 94.80% No DR, 4.45% NPDR+DME, 0.75% PDR. Study sub-sample proportions in N=215 participants in the UK were; 32.1% No DR, 24.7% NPDR + DME and 43.3% PDR and in N=208 participants in India were; 25.5% No DR, 25.0% NPDR + DME, 49.5% PDR.
Cystatin C and LRG1 were log-transformed, hence coefficients presented per 10% increase in pg/ml and ng/ml, respectively.
Missing data in HbA1c, age, duration, ethnicity (in the UK), and the variable under consideration were dropped for the models.

© 2022 Gurudas S et al. *JAMA Ophthalmology.*

eFigure 4. Sensitivity Analysis—ROC Curves Showing Discriminatory Ability With Serum Creatinine Instead of Cystatin C for UK[a] and India[b]

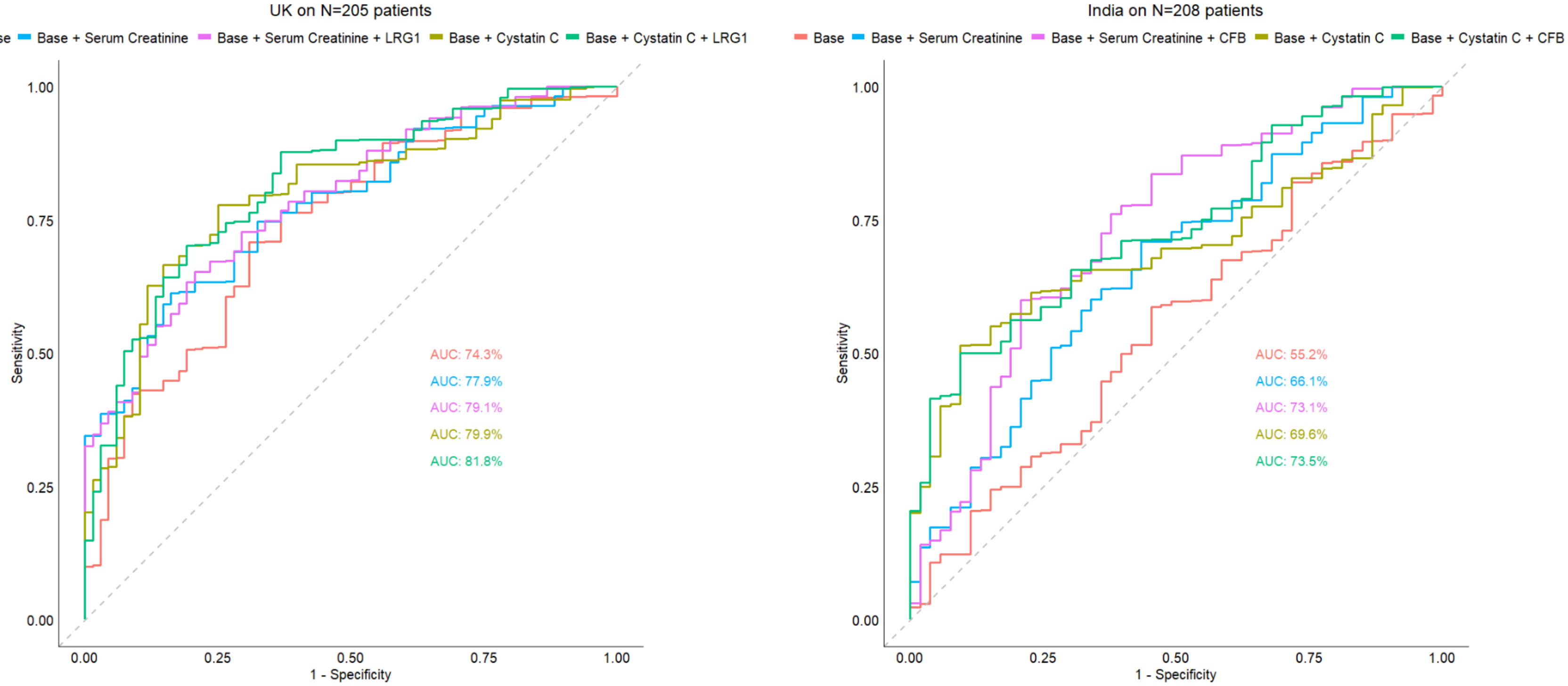

Abbreviations: DR, diabetic retinopathy; STDR, sight threatening diabetic retinopathy; LRG1, Leucine Rich Alpha-2-Glycoprotein 1 ; CFB, Complement factor B
Groups ii) No DR with Type-2 diabetes for at least 5 years and STDR groups iii) and iv) were used for statistical modelling.

[a] In the UK, population proportions; 93.96% No DR, 5.28% NPDR+DME, 0.76% PDR and in N=205 participants, sample proportion were 33.17% No DR, 23.90% NPDR +DME and 42.93% PDR, used to derive weights for the weighted logistic regression models. The base model includes age, diabetes duration, Ethnicity (Black, South Asian, Other) and HbA1c. Serum creatinine was included as a log-transformed variable. For comparison Base + Cystatin C (log) and Base + Cystatin C (log) + LRG1 (log) were plotted in this sample which contains patients with data on serum creatinine (N=205).

[b] In India, population proportions were; 94.80% No DR, 4.45% NPDR+DME, 0.75% PDR and in N=208 participants in India sample proportions were; 25.48% No DR, 25.00% NPDR + DME and 49.52% PDR, used to derive weights for the weighted logistic regression models.

The base model includes age, diabetes duration and HbA1c. As with the analysis on UK samples, serum creatinine was included as a log-transformed variable and for comparison Base + Cystatin C (log) and Base + Cystatin C (log) + CFB were plotted in this sample which contains patients with data on serum creatinine (N=208).

© 2022 Gurudas S et al. *JAMA Ophthalmology.*

eReferences

1. Lumley T, Scott A. Two-sample rank tests under complex sampling. *Biometrika.* 2013;100.

© 2022 Gurudas S et al. *JAMA Ophthalmology.*
